# Technique of cavoatrial tumor thrombectomy without cardiopulmonary by-pass

**DOI:** 10.1590/S1677-5538.IBJU.2019.0076

**Published:** 2020-08-10

**Authors:** Bhushan Patil, Nikhar Jain, S. K. Patwardhan, Amit Bellurkar

**Affiliations:** 1 Department of Urology KEM Hospital Mumbai India Department of Urology, KEM Hospital, Mumbai, India

## Abstract

**Introduction:**

Open surgery for tumor thrombi in atria is very challenging and are associated with significant morbidity and mortality rates. Here, we explore safety of foleys catheter assisted-technique, obviating the need for open surgery.

**Material and Methods:**

We performed Radical nephrectomy via the midlineincision for renal cell carcinoma with tumor thrombus extending into the right atrium. CTVS team was kept in standby all the time. Intra-operative ECHO was used for monitoring any migration of thrombi into pulmonary. Vessels.

**Results:**

Mean duration of surgery was roughly 4 hours. The time of total IVC occlusion was 2 minutes. The total blood loss was 2350 ml. Intraoperative ECHO showed complete removal of tumor thrombi.

**Conclusions:**

This procedure can be performed in high risk patients with solitary large tumor thrombi.

